# Benefits of Psychological Androgyny in Adolescence: The Role of Gender Role Self-Concept in School-Related Well-Being

**DOI:** 10.3389/fpsyg.2022.856758

**Published:** 2022-05-19

**Authors:** Selma Korlat, Julia Holzer, Marie-Therese Schultes, Sarah Buerger, Barbara Schober, Christiane Spiel, Marlene Kollmayer

**Affiliations:** ^1^Department for Psychology of Development and Education, Faculty of Psychology, University of Vienna, Vienna, Austria; ^2^Institute for Implementation Science in Health Care, University of Zurich, Zurich, Switzerland

**Keywords:** well-being, hedonic, eudemonic, school, gender, androgyny, adolescence

## Abstract

It has been repeatedly shown that the extent to which individuals adopt stereotypically masculine and feminine traits in their self-concept impacts their health and well-being. This is especially important in adolescence, when developmental changes and social pressures to conform to stereotypical gender roles can affect psychological functioning. However, previous studies investigating relationship between gender role self-concept and well-being in adolescents focused mostly on general well-being rather than well-being in specific contexts. Given that school is one of the most important contexts for adolescents’ development and well-being, the aim of this study was to investigate differences between adolescents with different gender role self-concepts (masculine, feminine, androgynous and undifferentiated) in *school-related well-being*. In line with the new conceptualization of well-being uniting hedonic (pleasure attainment and pain avoidance) and eudemonic (self-actualization and having meaningful purpose in one’s life) approaches, the present study used a measure of school-related well-being encompassing five domains suggested in the EPOCH (Engagement, Perseverance, Optimism, Connectedness and Happiness) model as well as a superordinate well-being factor. A total of 999 Austrian adolescents (52.2% girls, *M*_age_ = 13.79, *SD*_age_ = 1.53) answered inventories assessing adolescents’ gender role self-concept (GRI-JUG) and school-related well-being (EPOCH-G-S). The results supported the androgyny model of well-being, showing clear advantages of having both positive masculine and feminine qualities in one’s self-concept for optimal levels of school-related well-being. In addition, our results indicated the strong importance of femininity in adolescence and the school context. Theoretical and practical implications are discussed.

## Introduction

Adolescence is seen as a key stage of development characterized by profound changes in the biological and psychosocial domains (Choudhury et al., 2006). In this period, individuals should develop the capabilities required to lead a happy, healthy, and productive life (Laski, 2015). According to gender intensification theory, it is during this same time that girls and boys develop increasingly differentiated gender role identities due to increased pressure to conform to stereotypical gender roles (Hill and Lynch, 1983). These two developmental processes are interrelated, as it has been repeatedly shown that differences in the extent to which individuals adopt stereotypically masculine and feminine traits in their self-concept impact their psychological adjustment and well-being (Abele, 2014; Wang, 2016; Martínez-Marín and Martínez, 2019; Matud et al., 2019). Likewise, high levels of well-being in adolescence enable young people to deal with developmental tasks and promote a healthy transition to adulthood (Pyhältö et al., 2010). It has been shown that adolescents’ well-being is related to their academic functioning (Lewis et al., 2011), academic achievement (Berger et al., 2011), and is a protective factor for health in general (Carver et al., 2010). Studies have shown that adolescents with high levels of well-being are more resilient (Gilman and Huebner, 2006; Antaramian et al., 2010), have fewer depressive and anxiety symptoms, higher self-esteem, higher self-efficacy and higher adaptation (McKnight et al., 2002; Antaramian et al., 2010). These adolescents show enhanced mental and physical health outcomes and higher general life satisfaction (Tian et al., 2014).

Well-being in adolescence is integrally shaped by the everyday contexts in which adolescents grow and develop (Žukauskienė, 2014). As the place where adolescents spend almost one-third of their lives, school is one of the most important contexts within which adolescents’ development, including their well-being and gender socialization, unfolds (Eccles, 2004). It has been shown that adolescents’ experiences and relationships at school have an important impact on their perceived quality experiences and relationships in school of life (Jourdan et al., 2008) and likely have important implications for their lifelong development (Park, 2004; Eccles and Roeser, 2011). Given the developmental milestones related to gender identity and well-being in adolescence, as well as the importance of school in this period of life, this study aims to understand the role of gender role self-concept in well-being in the school context.

### Gender Role Self-Concept and Well-Being

Socialization pressure may lead adolescents to internalize societal gender role expectations as part of their self-concept (Bem, 1981; Hill and Lynch, 1983; Klaczynski et al., 2020). Gender role self-concept refers to the degree to which persons adopt stereotypically feminine and masculine attributes in their self-descriptions (Wolfram et al., 2009). In most studies investigating gender role self-concept, self-perception of expressive traits (e.g., being kind, gentle, sensitive to others) is used to assess femininity, and self-perception of instrumental traits (e.g., being independent, competitive, strong) is used to assess masculinity (Bem, 1974, 1981; Spence, 1991). While the equating of instrumentality (or agency) with masculinity, and expressiveness (or communion) with femininity has previously been questioned and criticized (e.g., Pedhazur and Tetenbaum, 1979), a more recent principal component analysis confirmed their interchangeability, showing that these concepts can be equated on an operational level (Abele and Wojciszke, 2007). Similarly, previous studies investigating the role of gender role self-concept in well-being labeled these gendered dimensions interchangeably when referring to the same concepts—self-ascribed gender stereotyped traits (see, e.g., Yarnell et al., 2019). Hence, we use and interpret these terms synonymously. Regardless of labels, four types of gender role self-concepts can be formulated based on continuous scores on these dimensions. *Masculine individuals* perceive themselves as high on masculine and low on feminine traits, *feminine individuals* score high on feminine and low on masculine traits, *androgynous persons* rate themselves as high on both sets of traits, and *undifferentiated individuals* view themselves as low on both sets of traits. The interrelations between gender role self-concept and well-being have been widely studied, as it has been noted that individual differences in these two dimensions affect overall functioning and health (e.g., Abele, 2014; Martínez-Marín and Martínez, 2019; Matud et al., 2019). There are three different models explaining the relationship between gender role self-concept and well-being, namely the congruence model, the androgyny model and the masculinity model.

Traditionally, individuals’ psychological well-being was thought to be related to their successful adoption of gender-typical behaviors and traits (the congruence model, Markstrom-Adams, 1989; DiDonato and Berenbaum, 2011). This hypothesis has received some support with preadolescent samples. For instance, Carver et al. (2003) found that early adolescents who perceived themselves to be atypical members of their same-sex peer group reported distress over their peer relations. In another study, the same authors confirmed that feeling gender-typical was positively related to adolescents’ well-being, whereas feeling pressure to conform to gender stereotypes was found to have a negative influence (Yunger et al., 2004). These authors did not measure gender role self-concept as the self-ascription of gender-typical traits, but focused on a measure of gender identity based on adolescents’ feelings of same-gender typicality, which is composed of five different components: membership knowledge, gender typicality, gender contentedness, felt pressure for gender conformity and intergroup bias (Egan and Perry, 2001). Other studies using Egan and Perry’s (2001) measure have also found that the more adolescents feel same-gender typical, the greater their self-esteem and the fewer internalizing problems they have (Corby et al., 2007; Menon et al., 2013; Pauletti et al., 2017).

The androgyny model (Bem, 1974; Spence and Helmreich, 1979) posits that psychological well-being is maximized when one has an androgynous gender role self-concept, which encompasses a broad set of attributes and behavioral options that allow for flexible behavior and successful coping with different demands and life situations. Studies have found that women and men whose self-concept includes both masculine-instrumental and feminine-expressive characteristics have greater well-being (e.g., Wang, 2016; Matud et al., 2019). More recent studies using both—a new measure of gender identity (Egan and Perry, 2001; Pauletti et al., 2017) and self-ascribed gender typical attributes (Martínez-Marín and Martínez, 2019), showed benefits of androgyny for well-being, self-esteem and psychological adaptation of adolescents. However, the proposed relationship between androgyny and psychological well-being has been called into question by empirical findings claiming that it is the masculine component of androgyny that is most associated with both adolescents’ and adults’ well-being (the masculinity model; Whitley, 1985). Indeed, the vast majority of studies have found masculinity to be associated with subjective well-being and other self-report measures of psychological adjustment (e.g., Wolfram et al., 2009; Abele et al., 2016; Matud et al., 2019). Studies with adolescents have come to more heterogeneous results. In an old study with adolescents aged 11, 13 and 15 in the United States using the Children’s Sex Role Inventory (CSRI; Boldizar, 1991), masculinity was linked to lower rates of depressive symptoms, while femininity was not (Priess et al., 2009). Another cross-sectional study using a brief version of the Bem Sex Role Inventory (BSRI) with 12,287 Norwegian adolescents aged 12 to 20 found femininity to be modestly positively correlated with depressed mood, whereas no such correlation was obtained for masculinity (Wichstrøm, 1999). On the other hand, Helgeson and Palladino (2012) found that both masculinity and femininity, measured as agentic and communal traits, were associated with positive relationship and health outcomes among US adolescents, with femininity being a stronger predictor than masculinity. A recent longitudinal study with Chinese children and adolescents aged 6–11 using self-descriptive questionnaires containing instrumental and expressivity traits found that older children’s self-esteem was more related to instrumental than expressive traits, whereas younger children’s self-esteem was more related to expressivity than instrumentality (Chen et al., 2018). These authors argued that expressive traits and behaviors are relatively more important to younger children’s self-esteem due to the prominence of social goals at this age; conversely, instrumental traits and behaviors are relatively more important to older children’s self-esteem due to the increasing importance of performance-related goals. In sum, empirical evidence supports both the masculinity and androgyny models of well-being, but indicates the stronger importance of femininity in adolescence compared to adulthood.

### School-Related Well-Being

Well-being has also been operationalized in heterogeneous ways in existing studies, ranging from self-esteem measures (e.g., Carver et al., 2003), positive and negative affect and life satisfaction scales (e.g., Buchanan and Bardi, 2015), absence of depression (e.g., Priess et al., 2009) and psychological distress (Helgeson and Palladino, 2012), to various measures of adjustment such as low internalizing problems (e.g., Pauletti et al., 2017). On the whole, most previous measures focused on hedonic well-being, which defines well-being in terms of attaining pleasure and avoiding pain (Kahneman, 1999), and refer to well-being as an outcome. However, in recent years, with the emergence and growth of positive psychology, well-being has been reconceptualized in a way that includes eudemonic aspect as well. This aspect refers to self-actualization and having meaningful purpose in one’s life, defining well-being in terms of personal growth experience (Tian et al., 2014). This led to the conceptualization of well-being as consisting of both hedonic and eudemonic dimensions in terms of the full functioning of the person, referring to well-being as a process (Ryan and Deci, 2001). Against this backdrop, Seligman (2011) proposed a five-element model consisting of Positive Emotions, Engagement, Relationships, Meaning, and Accomplishment (PERMA). Applying the PERMA model to adolescents (Kern et al., 2015) led to the development of the EPOCH model of adolescent well-being (Kern et al., 2016), which likewise encompasses five domains: Engagement, Perseverance, Optimism, Connectedness, and Happiness. While optimism and happiness correspond to the hedonic aspect of well-being, other EPOCH dimensions capture eudemonic characteristics. Engagement refers to the capacity to become absorbed in and focused on activities and tasks. Perseverance reflects the ability to keep striving toward one’s goals despite encountering obstacles. Optimism is characterized by hopefulness and confidence about the future. Connectedness describes satisfying relationships and friendships, giving and receiving support to and from others. Happiness refers to having a generally positive mood and feeling content with one’s life (Kern et al., 2016). Thus, EPOCH covers a wide range of components associated with optimal functioning in adolescence, taking into account both hedonic as well as eudemonic aspects of well-being. Hence, it unites domains that have been studied individually or combined under the umbrella term “well-being” in different constellations.

Another change resulting from positive psychology is the emphasis on youth’s optimal well-being in specific contexts (Seligman and Csikszentmihalyi, 2000; Elmore and Huebner, 2010; Long et al., 2012). School is a key life context for school-aged children and adolescents and a place where they spend a great deal of time. As such, school plays an important role in every aspect of youth development—it shapes their identity, intellectual and cognitive growth, social relationships and psychological well-being (Park, 2004; Long and Huebner, 2014; Verhoeven et al., 2019). Moreover, school is a place where adolescents prepare for their future (Eccles and Roeser, 2011). Understanding adolescents’ overall functioning and well-being in school is of utmost importance. However, most existing scales for subjective school-related well-being encompassed one cognitive element linked to the individual’s life satisfaction and two affective—positive affect and negative affect (see Liu et al., 2016) or used indicators of adolescents’ hedonic well-being and functioning in school (e.g., global school satisfaction or achievement; see Kern et al., 2015 or Yang et al., 2018 for further discussion). These indicators are, however, not considered as components of adolescents’ psychological well-being that focus on subjective experience (Holzer et al., 2021). Accordingly, previous studies showed well-being to be an important but distinguishable correlate of other indicators of academic functioning (e.g., Howell, 2009; Steinmayr et al., 2018). In line with the new conceptualization of well-being uniting hedonic and eudemonic approaches, in the present study a new measure of school-related well-being has been used: Buerger et al. (2022) have adapted the EPOCH model to the school context, resulting in the EPOCH-School (i.e., EPOCH-S), and its corresponding measure in German language, the EPOCH-German-School (i.e., EPOCH-G-S). Thus, engagement and perseverance according to the EPOCH-S model refer to school tasks and activities. Optimism refers to positive expectations of future academic success and future experiences at school. Connectedness refers to positive relationships in school in general, whether with peers or teachers, and happiness refers to positive mood in school and satisfaction with school life. By explicitly focusing on eudemonic and hedonic aspects of well-being, the EPOCH-S model distinguishes itself from previous operationalizations of school well-being *via* general school-related emotions or outcomes, reflecting the full variety of adolescents’ functioning in the school context. Moreover, the psychological characteristics and processes as indicators of well-being construct in the EPOCH-S model allow to derive specific intervention needs that might promote more global outcomes such as achievement or school satisfaction.

### Objectives of the Present Study

This study focuses on the relations between adolescents’ gender role self-concept and school-related well-being in terms of the EPOCH-S model. We investigate differences between adolescents with different gender role self-concepts (masculine, feminine, androgynous and undifferentiated) in terms of overall school-related well-being (EPOCH-S) as well as in the individual EPOCH-S dimensions: Engagement, Perseverance, Optimism, Connectedness, and Happiness.

Taking into account both social and performance-related challenges and requirements in school and the fact that androgynous individuals have the broadest repertoire of traits and behaviors (e.g., Bem, 1981; Pauletti et al., 2017), we expect androgynous boys and girls to show the highest levels of overall school-related well-being compared to the other three types of gender role self-concept. Similarly, we expect androgynous boys and girls to have the highest levels of the two hedonic dimensions—optimism and happiness. We have differential hypotheses for the eudemonic dimensions. Engagement and perseverance are instrumental qualities, as they reflect orientations toward achievement-related goals, while connectedness reflects the core value of expressivity—orientation towards others and social-related goals (Abele et al., 2016). Therefore, we expect masculine and androgynous boys and girls to exhibit the highest levels of engagement and perseverance and feminine and androgynous boys and girls to exhibit the highest levels of connectedness. Undifferentiated adolescents are expected to have the lowest levels of both overall school-related well-being as well as all individual dimensions compared to the other three types. We expected our hypotheses to be confirmed for both boys and girls of all ages in our study. In order to investigate potential changes in school-related well-being throughout middle adolescence, age was included as predictor in all analyses.

## Materials and Methods

### Participants and Procedure

The study sample consisted of 999 secondary school students (52.2% girls, M_age_ = 13.79, SD_age_ = 1.53; age range 12–17) from Vienna, Austria. Data collection took place in January 2020. To recruit participants, 10 secondary schools in Vienna were contacted by e-mail. Seven of these schools agreed to participate and were included in the sample. Students filled out paper-pencil questionnaires in their classrooms, supervised by trained research assistants. Participation in the study was voluntary and parental consent was obtained for participation in the study as well as data usage. The study was approved and supported by the local school board in accordance with Austrian federal law.

### Measures

#### Gender Role Self-Concept

To assess self-perceived femininity and masculinity, positive traits from the Inventory for Measuring Adolescents’ Gender Role Self-concept (GRI-JUG) were used (Krahé et al., 2007). Although the original GRI-JUG instrument encompasses negative traits as well, we assessed only positive traits in our study to keep the questionnaire’s length reasonable. This is in line with Bem’s theorizing (1981) and related studies (e.g., Woodhill and Samuels, 2003), showing that only positive androgyny, that is a balance of positive masculine and positive feminine qualities, is relevant for psychological well-being. Participants were presented with five masculine attributes (humorous, courageous, sporty, companionable, and strong; *α* = 0.68) and five feminine attributes (emotional, romantic, industrious, sympathetic and empathic; *α* = 0.66), and were asked to rate to what extent each attribute is characteristic of them on a 5-point Likert scale ranging from 1 (not at all true) to 5 (completely true). Scores were calculated for masculinity and femininity separately. The median-split procedure adopted by Spence et al. (1975) and Bem (1977) was used to determine the four types of gender role self-concept (see Table 1). Participants were classified into a 2 × 2 table according to whether they fell above or below the median score on the masculinity and femininity scales. Scores falling exactly on the median were classified as “high” scores (e.g., Carver et al., 2013). In the present sample, the median masculinity score was 3.8 and the median femininity score was 3.6. All four types significantly differed in both masculinity [*F*(3,993) = 681.094, *p* < 0.001] and femininity [*F*(3,993) = 669.293, *p* < 0.001], with androgynous adolescents scoring highest on both dimensions (*M* = 4.36 for masculinity and *M* = 4.12 for femininity), followed by masculine (*M* = 4.19) and feminine (*M* = 3.30) type on masculinity and feminine (*M* = 3.96) and masculine (M = 3.07) type on femininity. Undifferentiated type scored significantly lowest on both dimensions (M = 3.06 for masculinity and *M* = 2.85 for femininity) compared to other types.

**Table 1:** Gender role self-concept types.

		Femininity	
		Low	High
Masculinity	Low	Undifferentiated	Feminine
	High	Masculine	Androgynous

Low = scores smaller than the median, high = scores higher than the median.

#### Well-Being

School-related well-being was assessed with the EPOCH-G-S Measure of School-related Adolescent Well-Being (Buerger et al., 2022), a 19-item measure developed for students aged 10–18. The EPOCH-G-S measure of student’s well-being in school was validated with results favoring a second-order model with well-being as a second-order factor and the five specific EPOCH-S first-order factors. Invariance analyzes showed scalar invariance, indicating that factor means can be compared between boys and girls as well as between different age groups. Thus, the EPOCH-G-S with its multidimensional structure allows for detecting strengths and weaknesses in students’ well-being profiles and intervene on the school, class or individual level. The measures’ scales address the five dimensions of the EPOCH-S model: Engagement (four items, e.g., “When I do an activity for school, I enjoy it so much that I lose track of time”), Perseverance (four items, e.g., “When I have started a school task, I finish it”), Optimism (three items, e.g., “I am optimistic about my future at school”), Connectedness (four items, e.g., “When something good happens to me, I have people at school who I like to share the good news with”), and Happiness (four items, e.g., “I feel happy at school”). The measure uses a 5-point response format (1 = not true at all; 5 = completely true). The internal reliability of the EPOCH-G-S was *α* = 0.86. Reliabilities for the EPOCH-G-S subscales were *α* = 0.72 for Engagement, *α* = 0.79 for Perseverance, *α* = 0.67 for Optimism, *α* = 0.73 for Connectedness, and *α* = 0.85 for Happiness.

## Results

### Overall School-Related Well-Being

In order to examine differences in overall school-related well-being in adolescents, a 4×2 ANCOVA was conducted with gender role self-concept and sex as between-subject factors and age as a covariate. The mean score of all EPOCH-G-S items was the dependent variable. Means and standard deviations for overall school-related well-being by gender role self-concept and sex are presented in Table 2.

**Table 2:** Means and standard deviations for overall school-related well-being and single EPOCH-S dimensions by gender role self-concept and sex.

Gender role self-concept	Sex	*N*	Overall well-being		Engagement		Perseverance		Optimism		Connectedness		Happiness	
			*M*	*SD*	*M*	*SD*	*M*	*SD*	*M*	*SD*	*M*	*SD*	*M*	*SD*
Androgynous	Boys	163	3.71^a^	0.54	3.07^a^	0.85	3.81^a^	0.74	3.72^a^	0.81	4.15^a^	0.72	3.77^a^	0.87
	Girls	216	3.78^a*^	0.54	3.08^a*^	0.80	3.93^a*^	0.77	3.57^a^	0.79	4.34^a*^	0.68	3.86^a*^	0.86
Masculine	Boys	128	3.26^b^	0.53	2.61^b^	0.85	3.29^b,c^	0.85	3.25^b^	0.83	3.85^b^	0.68	3.32^b^	0.91
	Girls	58	3.54^b*^	0.53	2.79^b*^	0.74	3.67^b,c*^	0.83	3.41^b^	0.88	4.19^b*^	0.73	3.61^b*^	0.80
Feminine	Boys	53	3.32^b^	0.49	2.64^b^	0.75	3.60^b^	0.78	3.37^b^	0.74	3.76^b^	0.90	3.25^b^	0.93
	Girls	121	3.48^b*^	0.49	2.80^b*^	0.77	3.62^b*^	0.75	3.32^b^	0.82	4.15^b*^	0.77	3.47^b*^	0.84
Undifferentiated	Boys	133	3.11^c^	0.59	2.56^b^	0.89	3.49^c^	0.84	3.14^c^	0.88	3.51^c^	0.83	3.14^c^	0.89
	Girls	124	3.22^c*^	0.55	2.76^b*^	0.80	3.71^c*^	0.80	2.91^c^	0.80	3.76^c*^	0.86	3.18^c*^	0.84

Means not sharing the same (a, b, c) superscripts within column are significantly different, with superscripts from a to c indicating scores from highest to lowest. An * *indicates significant gender differences for each dimension with* * being placed next to the higher mean.

The results showed a significant effect of age, *F*(1, 996) = 27.72, *p* < 0.001, *η^2^p* = 0.027, indicating a negative relationship between age and school-related well-being, *r*(998) = −0.147, *p* < 0.001. There was also a significant main effect of gender role self-concept after controlling for adolescents’ age, *F*(3, 996) = 60.98, *p* < 0.001, *η^2^p* = 0.156. A Bonferroni *post hoc* test showed that androgynous adolescents (*M* = 3.73, *SD* = 0.54) reported significantly higher overall school-related well-being than masculine (*M* = 3.39, *SD* = 0.53), feminine (*M* = 3.42, *SD* = 0.49) and undifferentiated (*M* = 3.16, *SD* = 0.57) adolescents, all *ps* < 0.001. Undifferentiated adolescents, on the other hand, had significantly lower overall school-related well-being than adolescents with other gender role self-concepts, all *ps* < 0.01. There were no differences between masculine and feminine adolescents in overall school-related well-being, *p* > 0.05. The main effect of sex was also significant, *F*(1, 996) = 16.21, *p* < 0.001, *η^2^p* = 0.016, with girls (*M* = 3.50, *SD* = 0.53) reporting higher overall school-related well-being compared to boys (*M* = 3.35, *SD* = 0.54). The interaction between gender role self-concept and sex was not significant, *F*(3, 996) = 1.58, *p* = 0.192.

### Dimensions of School-Related Well-Being

To explore differences between boys and girls with different gender role self-concepts in the EPOCH-G-S dimensions, a two-way multivariate analysis of covariance (MANCOVA) with gender role self-concept and sex as between-subject factors and age as a covariate was conducted. The mean scores of the five EPOCH-G-S dimensions Engagement, Perseverance, Optimism, Connectedness and Happiness served as dependent variables in the model. Correlation coefficients between EPOCH-G-S dimensions are reported in Table 3. The MANCOVA yielded significant multivariate effects for gender role self-concept, *F*(3, 996) = 12.86, *p* < 0.001, *η^2^p* = 0.061, and sex, *F*(1, 996) = 11.76, *p* < 0.001, *η^2^p* = 0.056, as well as the covariate age, *F*(1, 996) = 14.30, *p* < 0.001, *η^2^p* = 0.068. The interaction effect between factors was not significant, *F*(3, 996) < 1, *p* = 0.476. We followed up on the significant multivariate effects with univariate analyses of covariance (ANCOVAs). Table 2 presents the means and standard deviations for all five EPOCH-G-S dimensions by gender role self-concept and sex.

**Table 3:** Correlations between EPOCH-S dimensions.

	1	2	3	4	5
Engagement	1	0.553^**^	0.341^**^	0.076^*^	0.349^**^
Perseverance		1	0.426^**^	0.141^**^	0.390^**^
Connectedness			1	0.274^**^	0.584^**^
Optimism				1	0.425^**^
Happiness					1

* *p < 0.05;*

** *p < 0.01.*

### Engagement

The results showed a significant effect of age, *F*(1, 996) = 16.88, *p* < 0.001, *η^2^p* = 0.017. There was a negative relationship between age and engagement, *r*(998) = −0.127, *p* < 0.001. There was also a significant main effect of gender role self-concept after controlling for age, *F*(3, 996) = 17.46, *p* < 0.001, *η^2^p* = 0.050. A Bonferroni *post hoc* test revealed that androgynous adolescents (*M* = 3.07, *SD* = 0.82) reported significantly higher scores on engagement than masculine (*M* = 2.69, *SD* = 0.82), feminine (*M* = 2.74, *SD* = 0.76), and undifferentiated (*M* = 2.65, *SD* = 0.85) adolescents. Differences among the other types were not significant, al *ps* > 0.05. The main effect of sex was also significant after controlling for age, *F*(1, 996) = 5.58, *p* < 0.05, *η^2^p* = 0.006, with girls (*M* = 2.86, *SD* = 0.80) reporting higher engagement than boys (*M* = 2.72, *SD* = 0.88).

### Perseverance

The results showed a significant effect of age, *F*(1, 996) = 38.28, *p* < 0.001, *η^2^p* = 0.037. The relationship between age and perseverance was negative, *r*(998) = −0.181, *p* < 0.001. The results showed also a significant main effect of gender role self-concept after controlling for age, *F*(3, 996) = 29.23, *p* < 0.001, *η^2^p* = 0.082. A Bonferroni *post hoc* test revealed that androgynous adolescents (*M* = 3.87, *SD* = 0.76) reported significantly higher scores on perseverance than masculine (*M* = 3.46, *SD* = 0.85), feminine (*M* = 3.64, *SD* = 0.76), and undifferentiated (*M* = 3.31, *SD* = 0.81) adolescents, *p* < 0.05. Undifferentiated adolescents reported lower scores than androgynous and feminine adolescents, *p* < 0.05. Mean differences between the other groups were not significant, *p* > 0.05. The main effect of sex was also significant after controlling for age, *F*(1, 996) = 10.40, *p* < 0.01, *η^2^p* = 0.010, with girls (*M* = 3.66, *SD* = 0.80) reporting higher perseverance than boys (*M* = 3.48, *SD* = 0.84).

### Optimism

The effect of age was not significant for optimism, *F*(1, 996) = 1.78, *p* = 0.182. The results showed a significant main effect of gender role self-concept, *F*(3, 996) = 29.47, *p* < 0.001, *η^2^p* = 0.082. A Bonferroni *post hoc* test showed that androgynous adolescents (*M* = 3.65, *SD* = 0.81) reported significantly higher scores on optimism than masculine (*M* = 3.33, *SD* = 0.85), feminine (*M* = 3.35, *SD* = 0.79), and undifferentiated (*M* = 3.02, *SD* = 0.85) adolescents, *p* < 0.05. Undifferentiated adolescents reported lower scores than all other groups, while there was no significant difference between masculine and feminine adolescents. The main effect of sex was not significant, *F*(1, 996) = 1.32, *p* = 0.251.

### Connectedness

The results showed no significant effect of age for connectedness, *F*(1, 996) < 1, *p* = 0.631. The main effect of gender role self-concept was significant, *F*(3, 996) = 32.73, *p* < 0.001, *η^2^p* = 0.090. A Bonferroni *post hoc* test revealed that androgynous adolescents (*M* = 4.24, *SD* = 0.70) reported significantly higher scores on connectedness than masculine (*M* = 4.02, *SD* = 0.71), feminine (*M* = 3.96, *SD* = 0.83), and undifferentiated (*M* = 3.64, *SD* = 0.85) adolescents, *p* < 0.05. Undifferentiated adolescents reported lower scores than all other groups, while there was no significant difference between masculine and feminine adolescents on connectedness. The main effect of sex was also significant, *F*(1, 996) = 30.60, *p* < 001, *η^2^p* = 0.030. Girls (*M* = 4.11, *SD* = 0.78) scored significantly higher on connectedness than boys (*M* = 3.82, *SD* = 0.80).

### Happiness

The effect of the covariate age was significant for happiness, *F*(1, 996) = 31.54, *p* < 0.001, *η^2^p* = 0.031. There was a negative relationship between age and happiness, *r*(998) = −0.167, *p* < 0.001. The main effect of gender role self-concept was also significant after controlling for age, *F*(3, 996) = 31.74, *p* < 0.001, *η^2^p* = 0.088. A Bonferroni *post hoc* test revealed that androgynous adolescents (*M* = 3.81, *SD* = 0.86) scored significantly higher on happiness than masculine (*M* = 3.45, *SD* = 0.88), feminine (*M* = 3.39, *SD* = 0.87), and undifferentiated (*M* = 3.15, *SD* = 0.87) adolescents, *p* < 0.05. Undifferentiated adolescents reported lower scores than all other groups, while there was no significant difference between masculine and feminine adolescents. The main effect of sex was significant after controlling for age, *F*(1, 996) = 7.37, *p* < 0.01, *η^2^p* = 0.007, with girls (*M* = 3.53, *SD* = 0.88) scoring higher on the happiness scale than boys (*M* = 3.37, *SD* = 0.93).

## Discussion

The goal of this study was to investigate relations between adolescent boys’ and girls’ gender role self-concepts and school-related well-being, taking into account both hedonic and eudemonic aspects of well-being. In general, our results support the androgyny model of well-being: androgynous boys and girls exhibited the highest levels of overall school-related well-being as well as the highest scores in all individual EPOCH-S dimensions: Engagement, Perseverance, Optimism, Connectedness, and Happiness. This finding is in line with Bem’s theorizing that individuals who score high in both masculinity and femininity display better adjustment and greater psychological health (Bem, 1981, 1993), as well as other studies confirming this notion in adolescent samples (Boldizar, 1991; Pauletti et al., 2017). School is the first social space after the home in which individuals experience obligations, engagement, commitment and relationships. Thus, having a broader set of attributes and behavioral options that allow for flexible behavior and successful coping with different demands is more important in school than anywhere else during adolescence. This might be even more important in secondary school when classes become more challenging, peer relationships grow more complex, and educational and professional goals are developed and shaped (Brown and Larson, 2009; Verhoeven et al., 2019). Our results also confirmed our assumption that undifferentiated adolescents exhibit the lowest scores in overall school-related well-being as well as all individual dimensions. Similar to androgynous persons, undifferentiated individuals are not gender-typed (Bem, 1977), but unlike androgynous individuals, they lack the enriched behavioral repertoire of androgynous persons and at the same time do not possess the positive characteristics typical of either a masculine or feminine self-concept. For that reason, the lowest levels of well-being were found in this group in previous studies focusing on general well-being (see Markstrom-Adams, 1989 for review) and were expected in the context of school-related well-being as well.

However, contrary to our expectations, androgynous girls and boys scored higher on all individual EPOCH-S dimensions compared to masculine and feminine girls and boys. This finding is not surprising for the two hedonic dimensions of school-related well-being—optimism and happiness—which refer to positive affect in the school context and, as such, are not related to either masculinity or femininity. However, the eudemonic dimensions—engagement, perseverance and connectedness—have more gendered connotations and encompass instrumental and expressive goals and behaviors. With engagement and perseverance being clearly achievement-oriented, and connectedness reflecting social-related goals, we expected masculine adolescents to achieve the same results as androgynous adolescents in engagement and perseverance, and feminine adolescents to be similar to androgynous adolescents on connectedness. The non-significant differences between adolescents with masculine and feminine gender role self-concepts in hedonic, but especially eudemonic dimensions, as well as in overall school-related well-being, speak in favor of the equal importance of masculinity and femininity for school-related well-being. Despite the clear dominance of the masculinity model over the femininity model of well-being in the literature (Wichstrøm, 1999; Priess et al., 2009; Abele et al., 2016), femininity seems to be as relevant as masculinity for adolescents’ functioning in school. This is not surprising giving the vital role of peer relationships in adolescence, as social interactions with classmates have been found to contribute to adolescents’ well-being (e.g., Sandstrom and Dunn, 2014). Moreover, feminine students are more liked by teachers and obtain better grades (Heyder and Kessels, 2013), have stronger school-related self-esteem and exhibit stronger feelings of belonging at school (Skinner et al., 2019). Thus, our results confirm previous studies indicating the stronger importance of femininity in this period and in the school context compared to later in life (Heyder and Kessels, 2013; Chen et al., 2018).

The salience of femininity for school-related well-being can be explained with William James’s theorizing that “self-centrality breeds self-enhancement,” according to which people’s judgement of their own self-worth is determined by the self-centrality of expressive or instrumental traits (Gebauer et al., 2013). Given the social nature of school, it can be argued that femininity occupies a more central position for adolescents’ school-related well-being than for general well-being or well-being in adulthood. On the other hand, schooling continually emphasizes performance- and achievement-related goals in the form of competence-related feedback, performance-based evaluations and expectations of adolescents to be successful and ambitious about their futures, making instrumentality integral to school-related well-being as well. Chen et al. (2018) argued that expressivity is relatively more central to younger children’s self-esteem due to their social goals, whereas instrumentality is relatively more central to older children’s self-esteem due to the importance of performance-related goals in that period of life. The effects of age on school-related well-being in our study did not confirm this. The results showed a negative relationship between age and the “instrumental” dimension of the EPOCH-S measure, with younger adolescents scoring higher on engagement and perseverance, whereas age did not have an effect on connectedness, indicating equal endorsement of items related to connectedness among adolescents of all ages. Although femininity might be more central for self-esteem at a younger age, connectedness in school seems equally important across adolescence from the age of 12 to age 17. On the other hand, engagement and perseverance as defined in the EPOCH-S model seem to be more endorsed by younger adolescents. This is not surprising given that adolescents’ behavioral and emotional involvement in academic activities declines as they grow older (Archambault et al., 2009). Our results also show a negative relationship between age and overall school-related well-being and happiness. Younger adolescents exhibit higher overall well-being and happiness. This finding is consistent with previous studies showing a decrease in overall well-being and positive affect from early to middle adolescence (Goldbeck et al., 2007; González-Carrasco et al., 2017).

Although one might expect stereotypical gender differences in the eudemonic EPOCH-S dimensions, our results showed that girls score higher in engagement, perseverance, connectedness, happiness, and overall school-related well-being than boys. There were no significant differences between boys and girls in optimism. In studies with adults, sex differences were found in gendered dimensions of well-being, with men scoring higher than women in self-acceptance and autonomy and women scoring higher than men in positive relations with others (Matud et al., 2019). A study applying the original EPOCH model of adolescents’ general well-being found small sex differences for optimism and connectedness only (Kern et al., 2016), with girls scoring higher on connectedness and boys on optimism. However, items assessing the EPOCH-S model are specific to the academic context, where girls exhibit higher engagement and tend to outperform boys (Duckworth and Seligman, 2006), which could explain girls’ higher endorsement of EPOCH-S items. More importantly, gender role self-concept has been shown to be a more important determinant of well-being and psychological adjustment than biological sex (e.g., Bem, 1993; Priess et al., 2009; Chen et al., 2018). Sex differences in this context are therefore secondary to the effects of gender role self-concept. Moreover, the interaction between sex and gender role self-concept was not significant for overall school-related well-being or for any individual dimensions, supporting Bem’s argument that androgyny does not offer more benefits to one gender than the other (Bem, 1993). In sum, our findings revealed that, beyond the effects of age and sex, androgynous adolescents experienced the highest levels of school-related well-being.

The results of this study have practical implications for school functioning. Developing curricular activities and a classroom environment that enhance both expressive and instrumental traits and behaviors in boys and girls may increase their school-related well-being. Interventions might focus on building performance- and achievement-related traits and goals as well as social-related traits and goals among all students, especially those at risk in terms of their well-being in school. These programs can not only contribute to school-related well-being among adolescents, but also decrease gender typing, which can result in reduced gender stereotypes in newer generations of adolescents.

### Limitations and Future Directions

Although the current study provides valuable insights into the relationship between gender role self-concept and school-related well-being, several limitations must be considered. First, this study focuses only on gender role self-concept, that is, the self-perceived possession of specific gender-stereotyped attributes, and does not take into account other facets of gender role identity. Future studies might investigate the relationship between androgyny and school-related well-being by assessing gender identity as overall felt gender typicality (see Egan and Perry, 2001). Second, although the effect sizes of gender role self-concept comparisons for overall well-being were moderate, the effect sizes for single EPOCH-S dimensions were small, limiting the practical relevance of the identified coefficients. Third, the inventory used to assess gender role self-concept in this study comprised only positive masculine and feminine attributes. Although it has been noted that *positive* androgyny results in higher psychological well-being (Woodhill and Samuels, 2003), future studies should investigate the relationship between negative gender-stereotyped attributes and well-being in the school context, which might provide new insights. Moreover, although in line with original instrument (Krahé et al., 2007), alpha reliabilities for both femininity and masculinity scale are rather low, indicating poor internal consistency of items constructing gender role self-concept types. However, scholars argue that constellations of gendered attributes similar to those used in our study reliably predict health and well-being (see, e.g., Abele, 2014; Yarnell et al., 2019). Future study thus should test similar design with another gender role identity measure for adolescent population. Fourth, this study does not address other factors impacting both gender socialization and school-related well-being. Further studies are needed to investigate the role of peers and teachers for the relation between gender role self-concept and school-related well-being. Finally, this study was conducted in a Western country and the results should not be generalized to other cultures. Androgyny seems to be beneficial for school-related well-being in individualist societies where traditional gender roles might be more liberal. In societies with more rigid gender norms or different cultural contexts, a different interplay between gender role self-concept and school-related well-being might be observed.

## Data Availability Statement

The datasets presented in this study can be found in online repositories. The names of the repository/repositories and accession number(s) can be found at: https://osf.io/5umnp/?view_only=2ea6ea1ceb61477192ea0f0fdb59aff1.

## Ethics Statement

Ethical review and approval was not required for the study on human participants in accordance with the local legislation and institutional requirements. Participation in the study was completely voluntary. Only those who gave active consent took part. Additionally, parental consent was obtained for participation in the study as well as data usage.

## Author Contributions

All authors listed have made a substantial, direct, and intellectual contribution to the work and approved it for publication.

## Conflict of Interest

The authors declare that the research was conducted in the absence of any commercial or financial relationships that could be construed as a potential conflict of interest.

## Publisher’s Note

All claims expressed in this article are solely those of the authors and do not necessarily represent those of their affiliated organizations, or those of the publisher, the editors and the reviewers. Any product that may be evaluated in this article, or claim that may be made by its manufacturer, is not guaranteed or endorsed by the publisher.
